# Development of Wideband Circular Microstrip Patch Antenna for Use in Microwave Imaging for Brain Tumor Detection

**DOI:** 10.3390/s26072062

**Published:** 2026-03-05

**Authors:** Hüseyin Özmen, Mengwei Wu, Mariana Dalarsson

**Affiliations:** 1 Electrical and Electronics Engineering Department, Dicle University, Diyarbakir 21280, Turkey; huseyin.ozmen@dicle.edu.tr; 2 Electrical Engineering Department, KTH Royal Institute of Technology, SE-100 44 Stockholm, Sweden; mengwei@kth.se

**Keywords:** microwave imaging, brain tumor, wideband antenna design

## Abstract

This work presents the design of a compact, wideband circular microstrip patch antenna for microwave imaging-based brain tumor detection. The main contribution is the development of a compact antenna structure incorporating enhanced ground-plane slot modifications, which significantly improves impedance bandwidth while maintaining a small electrical size, making it highly suitable for medical imaging systems. In addition, the study integrates antenna design, safety evaluation, and microwave imaging analysis within a unified framework to assess tumor localization feasibility using a realistic head model in CST Microwave Studio. The proposed antenna is fabricated on an FR-4 substrate with dimensions of 37 × 54.5 × 1.6 mm^3^, corresponding to an electrical size of 0.176λ × 0.260λ × 0.0076λ at the lowest operating frequency of 1.43 GHz. Ground-plane slot enhancements are introduced to achieve wideband performance, resulting in an impedance bandwidth from 1.43 to 4 GHz and a fractional bandwidth of 94.7%. The antenna exhibits a maximum realized gain of 3.7 dB. To evaluate its suitability for medical applications, specific absorption rate (SAR) analysis is performed using a realistic human head model at multiple antenna positions and at 1.5, 2.1, 2.5, 3.3, and 3.9 GHz frequencies. The computed SAR values range from 0.109 to 1.56 W/kg averaged over 10 g of tissue, satisfying the IEEE C95.1 safety guideline limit of 2 W/kg. For tumor detection assessment, time-domain simulations are conducted in CST Microwave Studio using a monostatic radar configuration, where the antenna operates as both transmitter and receiver at twelve angular positions around the head with 30° increments. The collected scattered signals are processed using the Delay-and-Sum (DAS) beamforming algorithm to reconstruct dielectric contrast maps and localize the tumor. It should be noted that the tumor-imaging demonstrations presented in this work are based on numerical simulations, while experimental validation is limited to the characterization of the fabricated antenna. Nevertheless, the findings indicate that the proposed antenna is a promising candidate for noninvasive, low-cost microwave brain tumor imaging applications.

## 1. Introduction

Brain cancer is one of the most devastating forms of cancer, with high mortality and limited treatment options. Glioblastoma multiforme (GBM), the most aggressive type, accounts for approximately 48% of all malignant brain tumors in adults [1]. According to the Global Cancer Statistics 2020, brain and central nervous system (CNS) cancers represent around 1.6% of all new cancer cases globally, but account for a disproportionately high mortality burden, with a 5-year relative survival rate below 35% in many countries. This underscores the critical need for early, non-invasive, and affordable diagnostic tools.

Microwave imaging (MWI) has attracted increasing attention as a potential medical diagnostic modality. It utilizes the dielectric contrast between healthy and malignant tissues, which affects how electromagnetic waves are scattered or absorbed. Unlike conventional imaging systems such as MRI (Magnetic Resonance Imaging) and CT (Computed Tomography), MWI offers advantages including low cost, portability, and the use of non-ionizing radiation [2]. It is particularly promising for continuous or point-of-care monitoring. MWI methods are typically divided into microwave tomography and radar-based imaging. The latter employs wideband time-domain pulses to detect dielectric discontinuities in biological tissue.

Radar-based MWI is preferable for portable systems because of its simple hardware, faster acquisition time, and robustness to noise [3]. In these systems, the antenna plays a pivotal role. In radar-based MWI, imaging performance is primarily influenced by antenna bandwidth, gain, directivity, group delay, and fidelity factor. A wide bandwidth is essential to achieve both sufficient penetration and high spatial resolution. The lower frequency limit affects penetration depth, while the upper frequency limit determines imaging resolution and the ability to detect small tumors. Antenna gain and directivity are critical for efficiently directing electromagnetic energy toward the target region, thereby improving signal-to-noise ratio and the strength of backscattered signals. Group delay stability is particularly important in time-domain systems, as variations cause pulse distortion. Similarly, a high fidelity factor ensures minimal waveform distortion, which is necessary for accurate tumor localization [4].

However, achieving high imaging performance often increases design complexity, cost, and system size, and must also comply with safety constraints such as specific absorption rate (SAR) limits. Therefore, an optimal imaging antenna should provide ultra-wideband performance, stable radiation characteristics, high gain, low signal distortion, and directed radiation toward the target region, while balancing imaging quality, cost-effectiveness, and patient safety.

Accordingly, a variety of wideband antennas have been proposed in recent studies for MWI applications. For example, a fern-shaped antipodal Vivaldi antenna (FAVA) demonstrated enhanced directivity and gain, improving brain tumor detection in near-field MWI [5]. A brick-shaped monopole antenna embedded in a semiflexible dielectric block was introduced to enhance detection sensitivity [6]. In another approach, a slotted patch antenna incorporating W-shaped and U-shaped radiating elements achieved wide bandwidth and reduced SAR, making it suitable for brain imaging applications [7]. A compact ellipse shaped patch antenna with ground slot was developed for head imaging, offering high gain while maintaining SAR safety limits [8]. In addition, a metamaterial-enhanced structure with split-ring resonators demonstrated improved impedance matching and signal detectability [9]. Furthermore, wideband slotted patch antennas with partial ground structures were proposed to enhance radiation efficiency in microwave-based head imaging systems [10].

Building upon these developments, this work presents a novel circular microstrip patch antenna with a slot-loaded ground plane optimized for radar-based microwave brain imaging applications. The proposed antenna operates over a wide frequency band of 1.43–4 GHz and is analyzed through full-wave electromagnetic simulations using CST Microwave Studio. The incorporation of a ground-plane slot alters the surface current distribution, resulting in improved impedance bandwidth and enhanced radiation characteristics. Owing to this design optimization, the antenna achieves a maximum realized gain of approximately 3.7 dBi within the operating band. Furthermore, the proposed antenna is designed as a compact and cost-effective solution by utilizing an FR-4 substrate, which is inexpensive and readily available. To ensure compliance with safety regulations, the SAR analysis is conducted using a realistic human head model at multiple antenna positions and at 1.5, 2.1, 2.5, 3.3, and 3.9 GHz. The calculated SAR values range from 0.109 to 1.56 W/kg averaged over 10 g of tissue, satisfying the IEEE C95.1 safety guideline limit of 2 W/kg. A realistic multilayer human head model with an embedded tumor is employed to evaluate the imaging performance of the proposed antenna. The tumor imaging capability of the antenna is evaluated entirely in a simulation environment, where spherical tumors with radii of 4 mm, 5 mm, 6 mm, 7 mm and 10 mm are placed at different locations within the brain tissue. Image reconstruction is performed using the Delay-and-Sum beamforming algorithm, and the results demonstrate accurate localization of the tumor. The imaging results indicate that tumors of varying sizes and positions can be detected with a high success rate, demonstrating the robustness and sensitivity of the proposed antenna for microwave-based brain imaging applications.

## 2. Materials and Methods

### 2.1. Antenna Design

Microstrip patch antennas are widely used in medical MWI due to their planar structure, ease of fabrication, low profile, and integration capability. However, a key limitation of conventional patch antennas is their narrow impedance bandwidth. To mitigate this limitation, a circular microstrip patch antenna with a slot-loaded ground plane is designed, aimed at enhancing both the bandwidth and the radiation characteristics.

The antenna was modeled and simulated using CST Studio Suite (v2021). It was designed to operate within the 1.43–4 GHz frequency band, which is suitable for brain tissue imaging, where a balance between penetration depth and spatial resolution is essential [11]. FR4 substrate was selected due to its low cost and wide availability, with a dielectric constant of ε_r_ = 4.3 and a thickness of 1.6 mm. The radiating part and ground plane is copper with 0.035 mm thickness.

**Figure 1:**
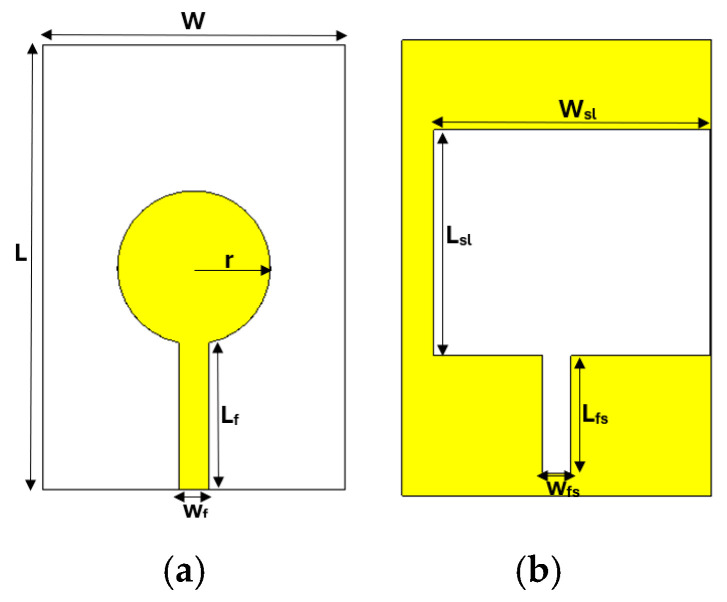
Designed microstrip antenna: (**a**) front view with circular patch; (**b**) back view with slot on the ground plane.

Figure 1 presents the front and back views of the proposed antenna. The front view shows the circular radiating patch, while the back view highlights the two rectangular slots etched into the ground plane to enhance performance.

The values of the geometrical parameters of the proposed antenna are presented in Table 1 as follows.

To improve bandwidth, a rectangular slot structure was etched into the ground plane. This technique increases the effective electrical length and introduces additional current paths, effectively generating multiple resonances. Such slot-loading methods have been employed successfully in recent works [12,13], and are conceptually similar to edge-slot enhancements in Vivaldi antennas [14].

#### Parameter Optimization

The reflection coefficient S_11_ is a key metric for evaluating antenna performance, representing the amount of power reflected back at the antenna input. A good design maintains S_11_ < −10 dB across the target bandwidth, indicating effective impedance matching and minimal return loss. To achieve this, a four-step iterative optimization was performed on the ground plane geometry:

- Step 1: Plain ground without slot served as baseline, exhibiting poor bandwidth and resonance.
- Step 2: Added central rectangular slot, which improved low-frequency impedance matching.
- Step 3: Added secondary slot underneath the main rectangle, which introduced additional resonance.
- Step 4: Introduced geometric asymmetry, resulting in wider bandwidth improved impedance matching.

**Figure 2:**
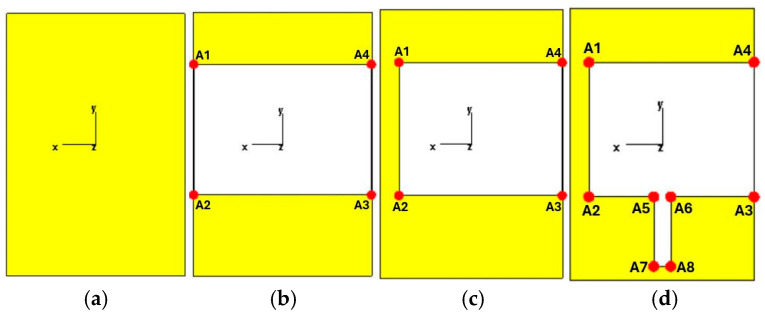
Evolution of the antenna ground design: (**a**) step 1; (**b**) step 2; (**c**) step 3; (**d**) step 4.

All evolution steps of the antenna ground slot is shown in Figure 2.

**Figure 3:**
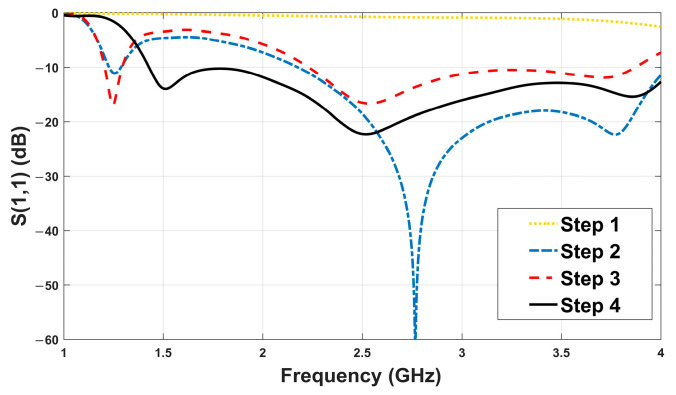
Simulated reflection coefficient (S_11_) for the four design steps. Step 4 demonstrates optimal wideband behavior.

As illustrated in Figure 2a, in the first design step, no slot is introduced in the ground plane of the antenna. In the second step (Figure 2b), a rectangular slot is etched in the ground plane with corner coordinates (x, y) A1 (18.4, 16.5), A2 (18.4, −10.5), A3 (−18.4, −10.5), and A4 (−18.4, 16.5). In the third step (Figure 2c), the slot geometry is modified by shifting the coordinates of A1 and A2 to (14.7, 16.5) and (14.7, −10.5), respectively, while the coordinates of A3 and A4 remain unchanged. In the fourth step (Figure 2d), the coordinates of A1, A2, A3, and A4 are kept identical to those in the third step, and an additional slot is introduced with corner coordinates A5 (1.7, −10.5), A6 (−1.7, −10.5), A7 (1.7, −24.5), and A8 (−1.7, −24.5). The introduction of asymmetry in Step 4 significantly improved the antenna’s frequency response. Figure 3 shows the S_11_ performance of the antenna for each step.

The final design (Step 4) achieves wideband impedance matching across the entire target range (1.43–4 GHz), meeting the requirements for radar-based MWI [15]. This improvement is achieved through a series of structural modifications, including the ground plane slotting and feed transition optimization, which progressively enhance impedance bandwidth and matching performance. The introduced geometric asymmetry in the ground slot significantly affects the antenna performance. The asymmetric structure modifies the surface current distribution and introduces additional current paths on the ground plane. As a result, multiple resonant modes are generated and coupled together, which leads to an extended impedance bandwidth. Furthermore, the gradual impedance transition created by the asymmetric slot improves the impedance matching over a wider frequency range.

**Figure 4:**
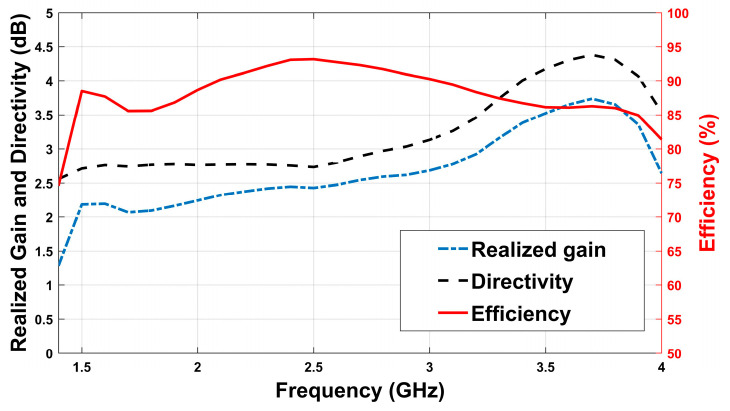
Simulated realized gain, directivity and efficiency of the proposed antenna versus frequency.

Figure 4 shows the realized gain, directivity and efficiency of the proposed antenna across 1.43–4.0 GHz. According to the figure, the realized gain increases steadily with frequency, peaking at approximately 3.7 dB near 3.6 GHz.

The directivity follows a similar trend, reaching a maximum of about 4.4 dB at the same frequency. Finally, the efficiency improves rapidly from just above 50% at 1.4 GHz to over 85% by 1.6 GHz, maintaining values above 85% up to 3.9 GHz, with a peak of 93% between 2.5–3.2 GHz. These results indicate that the antenna provides high efficiency and moderate gain over a broad bandwidth, making it suitable for MWI applications, where consistent performance and low-loss operation are critical.

**Figure 5:**
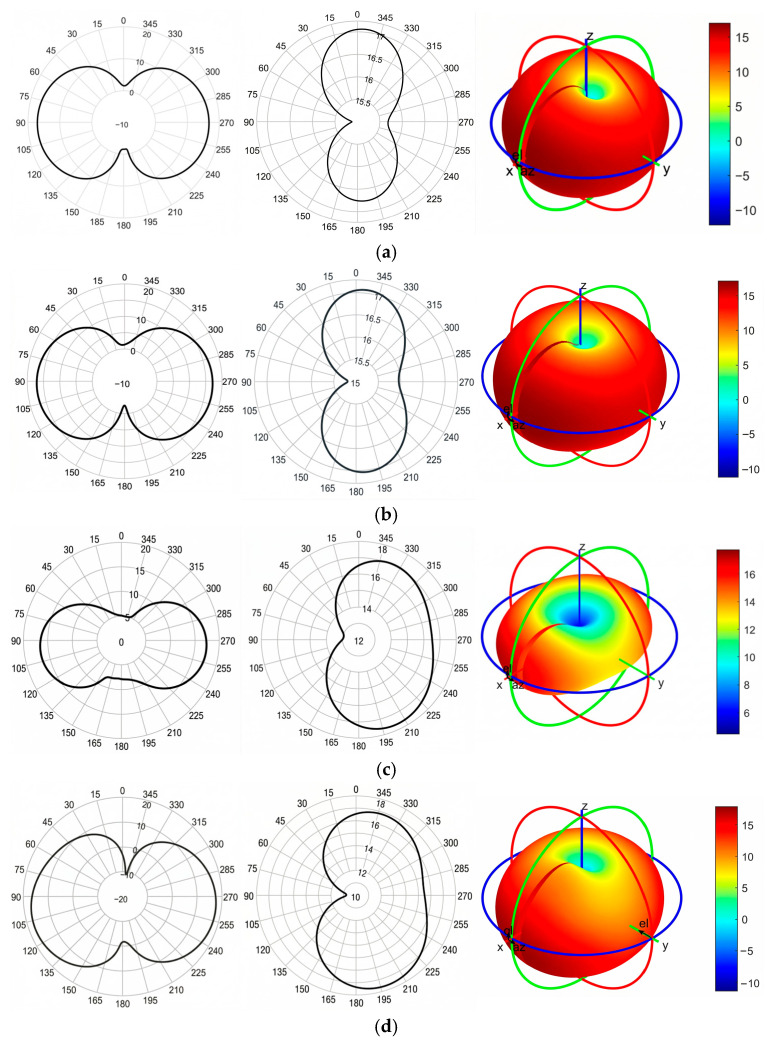
Simulated far-field radiation patterns of the proposed antenna. Each row shows E-plane, H-plane, and 3D radiation pattern for (**a**) 2.1 GHz; (**b**) 2.5 GHz; (**c**) 3.3 GHz and (**d**) 3.9 GHz frequencies.

To further validate the radiation performance of the proposed antenna across its operational band, we evaluated both 1D and 3D radiation patterns at four representative frequencies: 2.1 GHz, 2.5 GHz, 3.3 GHz, and 3.9 GHz. Figure 5 shows the E-plane (XY-plane), H-plane (XZ-plane), and 3D radiation patterns for each frequency.

As shown in Figure 5, the antenna exhibits stable directional radiation patterns across the operating band. The E-plane and H-plane patterns remain consistent and symmetric, with broad main lobes. The 3D far-field plots demonstrate upper-hemispheric radiation and confirm strong directivity performance. These results verify the antenna’s effectiveness for wideband MWI applications.

**Figure 6:**
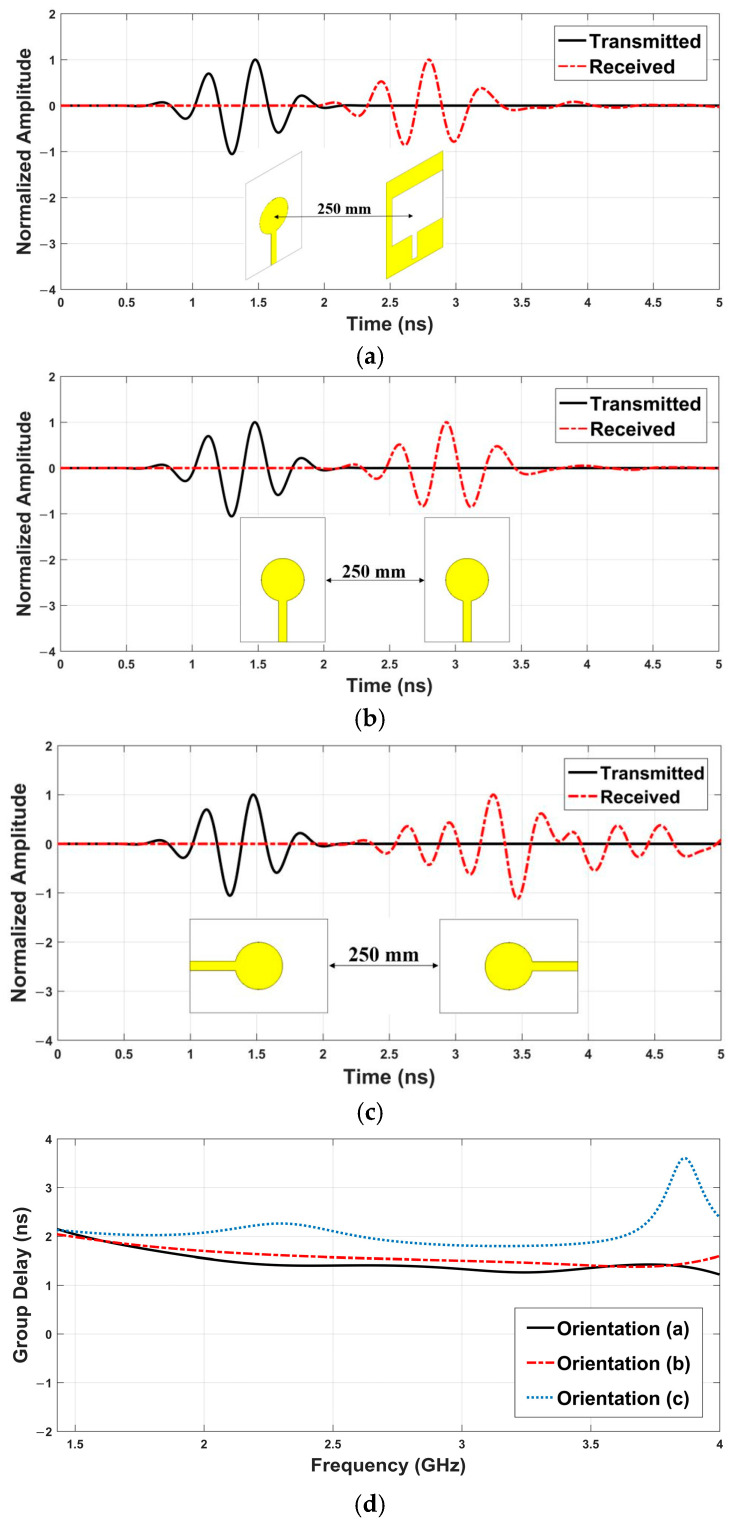
Transmitted and received waveforms in three different orientations (**a**–**d**). For clarity, the antenna orientations are included at the bottom of each figure.

In MWI for brain tumor detection, high waveform fidelity is essential for accurate reconstruction of reflected signals from tissue boundaries, while low and stable group delay is critical for maintaining signal timing and minimizing image artifacts. Figure 6 presents the transmitted and received waveforms for three antenna orientations (a–c) along with group delay characteristics (d).

The face-to-face orientation (Figure 6a) yields very high waveform fidelity at 95% and the lowest average group delay of 1.48 ns across the 1.43–4 GHz band, indicating minimal signal distortion and optimal timing. The side-by-side configuration (Figure 6b) shows a slightly degraded performance, with a group delay of 1.59 ns. The third case (Figure 6c) results in the highest distortion and a delay of 2.1 ns, implying greater dispersion. Group delay curves in Figure 6d confirm these findings, with the face-to-face setup offering the most consistent performance for radar-based MWI.

### 2.2. Head Modeling

#### 2.2.1. Head Model and Radar Configuration

**Figure 7:**
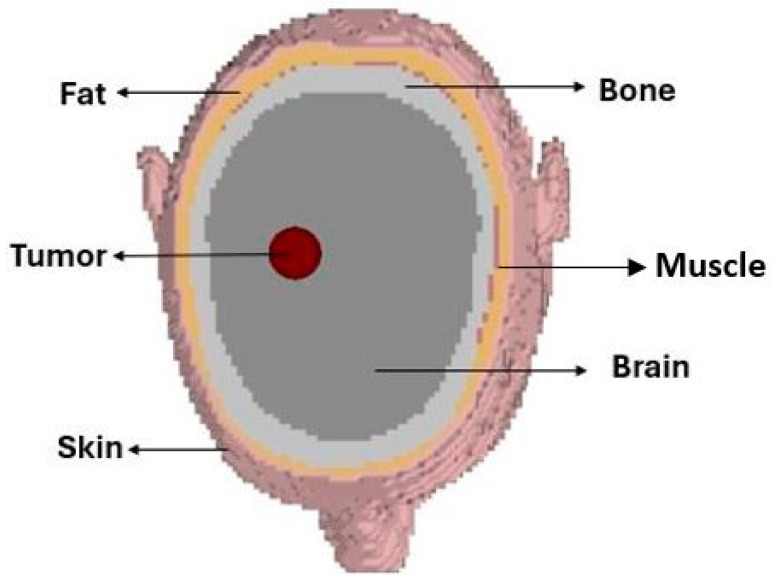
Gustav head model and tissue layers.

A crucial component in validating the imaging system is the development of a realistic human head phantom that accurately replicates both anatomical geometry and electromagnetic tissue properties. In this work, we utilized a voxel-based head model (Gustav) in CST, segmented into five primary layers: skin, fat, muscle, bone and brain, as shown in Figure 7.

In the operational band of 1.43–4 GHz, the gray and white matter exhibit an average relative permittivity of approximately ε_r_ ≈ 42 and conductivity of approximately σ ≈ 0.98 S/m. Tumor tissues generally contain higher water content, leading to increased permittivity and conductivity—values often exceeding ε_r_ ≈ 60 and σ ≈ 1.2 S/m [16]. This dielectric contrast is the physical basis for target localization in MWI systems.

**Figure 8:**
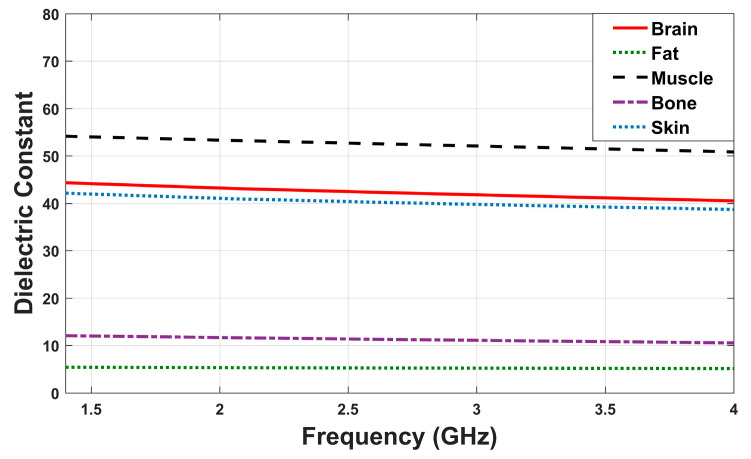
Relative permittivity (ε_r_) variation over simulated frequency.

Figure 8 illustrates the frequency-dependent variation in dielectric constant (ε_r_) of the tissues used in the simulation setup to more realistically model the antenna’s interaction with biological tissue.

**Figure 9:**
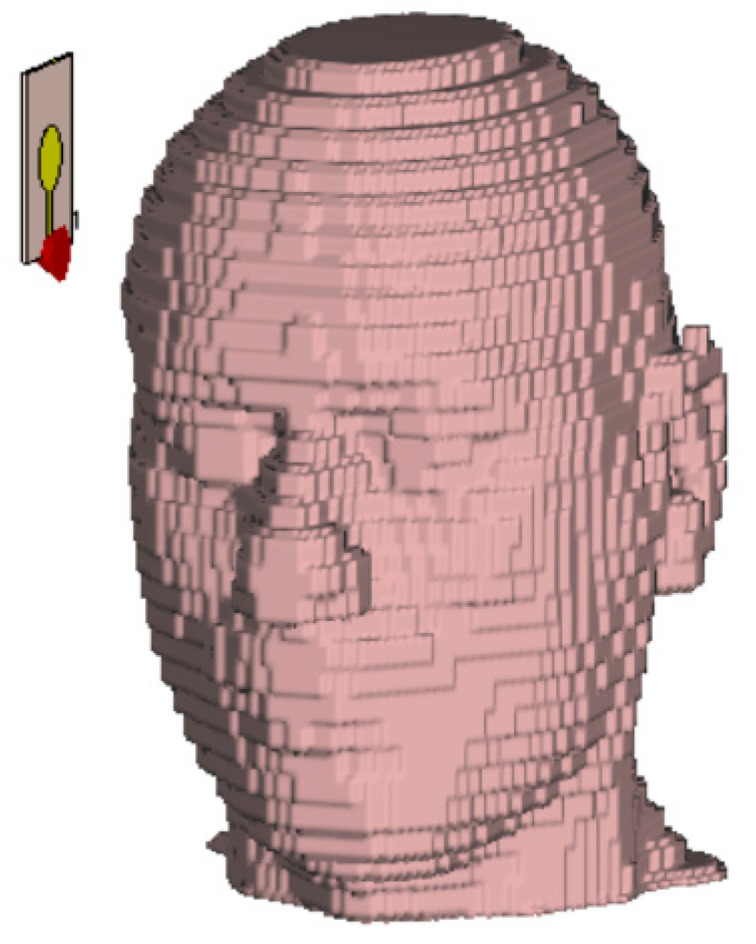
The antenna and the head phantom geometry in CST Studio.

Figure 9 shows the voxel-based human head phantom used within the CST Studio environment and the first position of the antenna.

**Figure 10:**
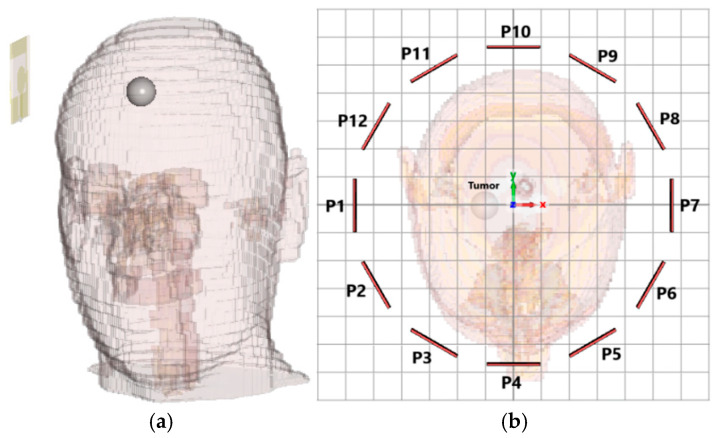
(**a**) Spherical tumor (r = 10 mm) embedded within the brain region of the head phantom, (**b**) antenna positions monostatically arranged around the head.

To simulate a tumor, a spherical dielectric inclusion (10 mm radius) was embedded within the parietal lobe region of the gray matter, as shown in Figure 10a. Figure 10b illustrates the monostatic measurement setup, where data is acquired from 12 different positions uniformly distributed around the head at 30-degree angular increments. In each position, the same antenna operates in monostatic mode, serving both as transmitter and receiver. This full 360-degree configuration ensures comprehensive coverage of the head, allowing for effective signal capture from multiple angles to improve imaging accuracy. The antenna positions are labeled P1 to P12 and are arranged to maintain a consistent distance from the head surface, targeting the suspected tumor region located near the center of the head model.

The parietal lobe was selected based on clinical reports identifying it as a common site for gliomas. Placing the tumor asymmetrically allows for validation of the system’s angular resolution and side-discrimination capabilities.

Two simulation conditions were defined:

- Case 1 (healthy baseline): The tumor was assigned same dielectric properties as the surrounding tissue (ε_r_ = 42, σ = 0.98 S/m).
- Case 2 (tumor present): The tumor region was assigned elevated dielectric properties to introduce contrast (ε_r_ = 60, σ = 1.2 S/m).

This configuration enables calibration of the signals by subtracting the baseline field distribution from the tumor-present scenario, isolating the tumor-induced responses. Such background-subtraction techniques are standard in radar-based MWI systems, enhancing the detectability of low-contrast inclusions that might otherwise be obscured by strong reflections from the skull or scalp.

#### 2.2.2. Electric Field Distrubition and SAR

Analyzing the electric field distribution within the brain is essential for understanding how electromagnetic waves interact with the imaging region across the operating frequency range, as it directly influences penetration depth, spatial resolution, and tumor detectability.

**Figure 11:**
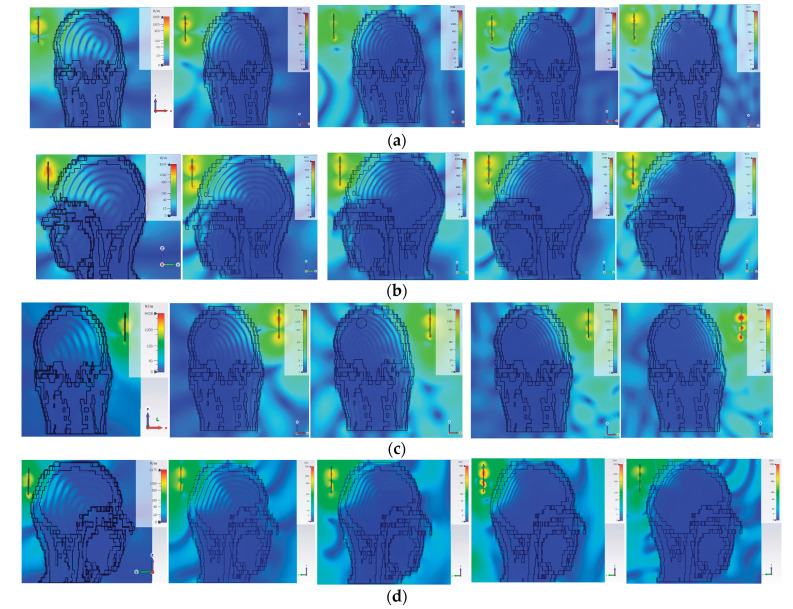
Electric field distrubution at 1.5, 2.1, 2.5, 3.3 and 3.9 GHz frequencies at the different antenna positions: (**a**) P1; (**b**) P4; (**c**) P7 and (**d**) P10, as indicated in Figure 10.

Figure 11 shows the electric field distribution in the head model at 1.5, 2.1, 2.5, 3.3 and 3.9 GHz for different antenna positions. Lower frequencies (e.g., 1.5 GHz) achieve deeper penetration, which is suitable for detecting tumors in inner brain regions, while higher frequencies (3.3–3.9 GHz) enhance spatial resolution, albeit with reduced penetration depth. The results confirm the trade-off between penetration depth and resolution, and highlight the importance of multi-frequency and multi-position antenna scanning to improve tumor detection accuracy.

**Figure 12:**
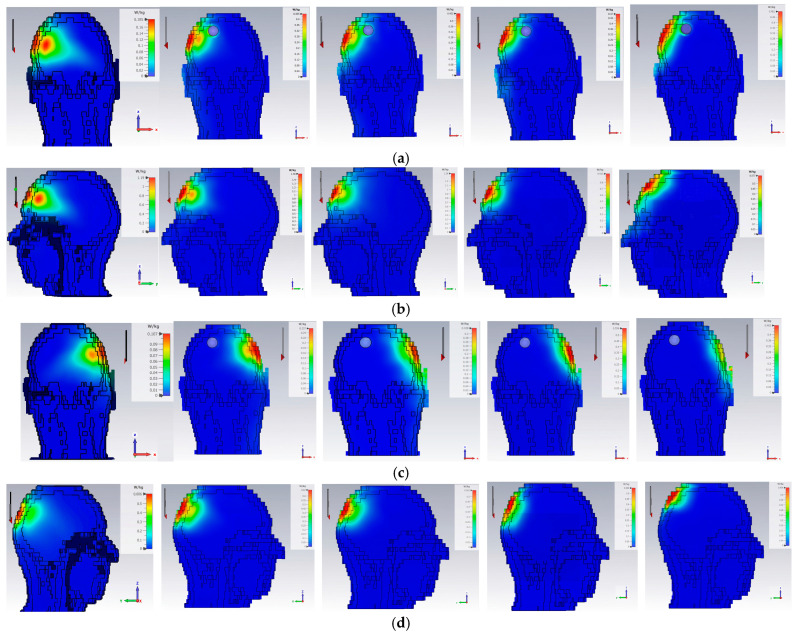
SAR results at 1.5, 2.1, 2.5, 3.3 and 3.9 GHz frequencies at the different antenna positions: (**a**) P1; (**b**) P4; (**c**) P7 and (**d**) P10, as indicated in Figure 10.

SAR quantifies the rate at which electromagnetic energy is absorbed per unit mass of tissue, and is a critical parameter for evaluating the safety of microwave-based medical imaging systems. Figure 12 and Table 2 presents the simulated SAR distribution and levels within the head model at frequencies of 1.5, 2.1, 2.5, 3.3, and 3.9 GHz for four different antenna positions (P1, P4, P7, and P10).

The results indicate that the maximum SAR values vary with both frequency and antenna position, ranging from 0.109 W/kg to 1.560 W/kg over 10 g of tissue. The highest SAR value (1.560 W/kg) occurs at 2.1 GHz in position 2, while other positions and frequencies remain well below this level. All obtained SAR values are within the IEEE C95.1 safety guideline limit of 2 W/kg averaged over 10 g of tissue [17], confirming that the proposed antenna design is safe for medical imaging applications. Additionally, the relatively low SAR levels across most frequencies and positions demonstrate that the system achieves sufficient electromagnetic field penetration while maintaining patient safety. These results confirm the suitability of the antenna for brain tumor detection using MWI.

### 2.3. Signal Processing and Imaging

**Figure 13:**
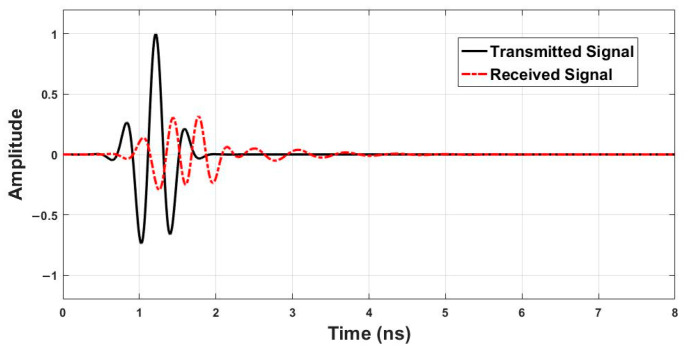
Transmitted and received time-domain signals.

To excite the antenna and probe internal dielectric variations, a Gaussian-modulated pulse was used as the input signal. This type of signal is commonly employed in ultrawideband radar systems due to its sharp temporal localization and wide frequency spectrum, providing both good resolution and deep penetration. Figure 13 displays an example of the transmitted and received time-domain waveforms at the P1 location. The reflected signal contains overlapping returns from various tissue boundaries and internal scatterers, including the tumor.

For full spatial coverage, the antenna is rotated around the head in 30° increments, producing 12 equidistant views (see Figure 10). At each angular position, both baseline (healthy) and tumor-present simulations are performed to enable background subtraction.

#### 2.3.1. Calibration and Beamforming

The collected time-domain responses contain strong reflections from interfaces such as the skin and skull, which can obscure weaker signals originating from intracranial anomalies like tumors.

To enhance tumor-specific features, a calibration technique was employed. In order to obtain the tumor responses, signals reflected from both the healthy brain and the brain with a tumor were collected for each channel. Then, the signals reflected from the healthy brain were subtracted from those reflected from the brain with a tumor. In this way, signals reflected from other tissue types were filtered out, and only the signals reflected from the tumor were obtained.

The calibrated signal at angular position m and time index n is computed as:

(1) $$S_{m}=X_{m}\left(withtumor\right)(n)-X_{m}(healthy)(n)$$

where $X_{m}(n)$ is the raw received signal at the m-th position, M = 12 is the total number of angular views, and $S_{m}(n)$ is the background-suppressed signal that isolates and emphasizes dielectric anomalies.

After the calibration process, tumor localization is achieved using the Delay-and-Sum (DAS) beamforming algorithm. The energy intensity at the spatial voxel position r is calculated by:

(2) $$I\left(n\right)=\int_{0}^{T}{\left[\sum_{m=1}^{M}S_{m}\left(t-\tau_{m}(r)\right)\right]}^{2}dt$$

The time delay $\tau_{m}\left(r\right)$ represents the round-trip propagation time between the m-th antenna and point r:

(3) $$\tau_{m}\left(r\right)=\;\frac{2d_{m}}{v}$$

with $d_{m}$ denoting the Euclidean distance:

(4) $$d_{m}=\sqrt{{\left(x-x_{m}\right)}^{2}+{\left(y-y_{m}\right)}^{2}\;{+\;(z-z_{m})}^{2}}$$

and v being the wave speed in brain tissue, approximated as:

(5) $$v=\frac{c}{\sqrt{\varepsilon r}}$$

where c is the speed of light in vacuum. The DAS algorithm aligns and coherently sums the delayed backscatter waveforms, reinforcing energy from true dielectric scatterers such as tumors while suppressing noise and anatomical clutter. The output is a spatial intensity map that enables visual localization of the tumor region with high contrast.

#### 2.3.2. Imaging Results

**Figure 14:**
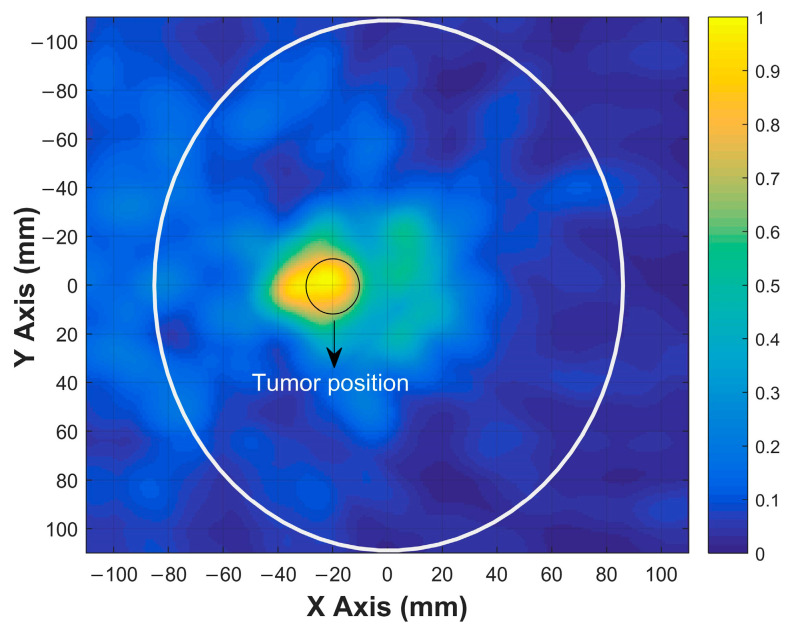
Reconstructed 2D image of the tumor in the brain.

Using the calibrated signals from the 12 antenna positions indicated in Figure 10, a 2D image was reconstructed based on the DAS beamforming algorithm described in the previous section. The calibrated time-domain signals were aligned and coherently summed using delay profiles derived from the assumed propagation velocity in the brain tissue. The reconstructed image highlights the dielectric contrast introduced by the tumor region. As shown in Figure 14, a clear energy peak appears at the expected location of the tumor, confirming the effectiveness of the imaging system.

This result demonstrates that the proposed antenna design and imaging method can successfully localize high-permittivity tumors in brain tissue.

**Figure 15:**
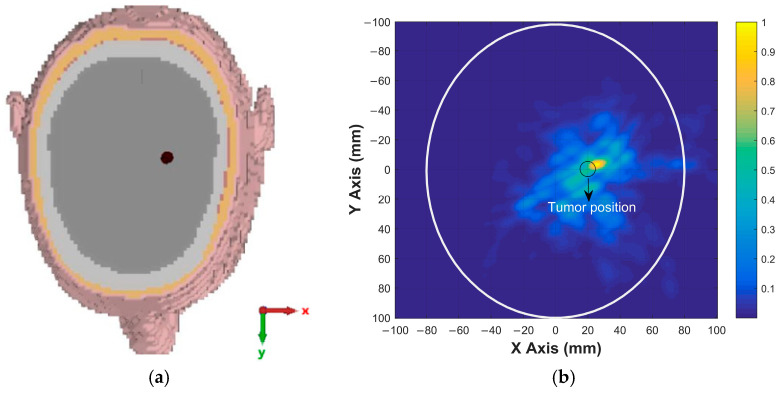
(**a**) Position of 4 mm radius tumor in the brain; (**b**) the image of the tumor on the x–y plane.

**Figure 16:**
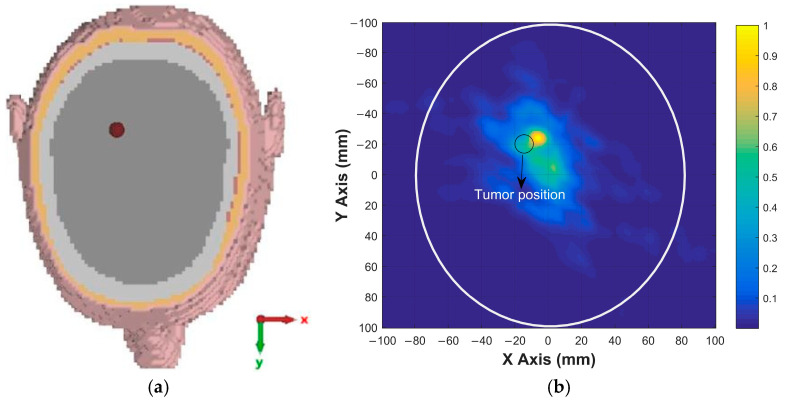
(**a**) Position of 5 mm radius tumor in the brain; (**b**) the image of the tumor on the x–y plane.

**Figure 17:**
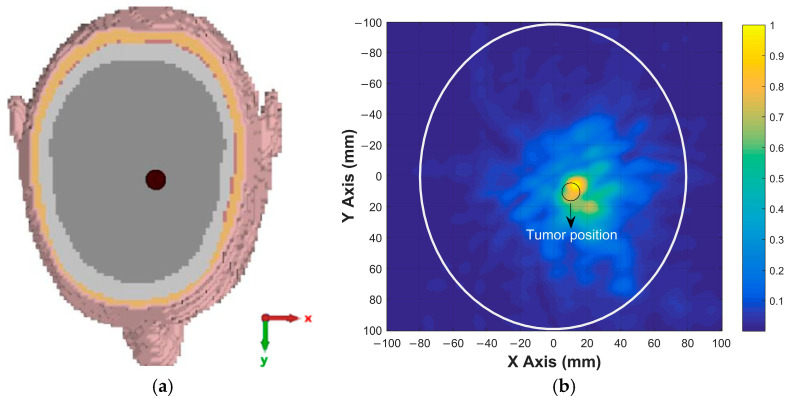
(**a**) Position of 6 mm radius tumor in the brain; (**b**) the image of the tumor on the x–y plane.

**Figure 18:**
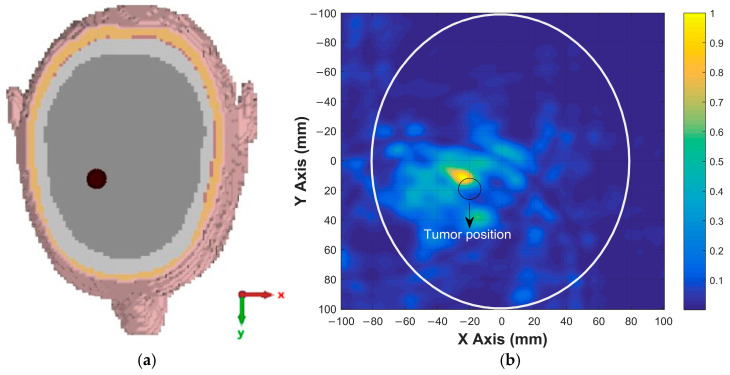
(**a**) Position of 7 mm radius tumor in the brain; (**b**) the image of the tumor on the x–y plane.

To further evaluate the tumor detection capability of the proposed antenna, simulations were carried out for tumors with different sizes and locations. These simulations were performed to assess the ability of the antenna and imaging system to detect tumors of varying dimensions and positions within the brain. For a tumor with a radius of 4 mm located at (20, 0), the x–y cross-sectional view of the head model is shown in Figure 15a, while the corresponding reconstructed tumor image obtained after the simulation and signal processing stages is presented in Figure 15b. For a tumor with a radius of 5 mm located at (−15, −20), similar cross-sectional and reconstructed images are shown in Figure 16. The imaging results for a tumor with a radius of 6 mm located at (10, 10) are presented in Figure 17, whereas the reconstructed image corresponding to a tumor with a radius of 7 mm located at (−20, 20) is shown in Figure 18. These results demonstrate that the proposed antenna is capable of successfully detecting small-sized tumors located at different positions within the brain, confirming its effectiveness for microwave-based brain tumor imaging applications.

## 3. Experimental Results

**Figure 19:**
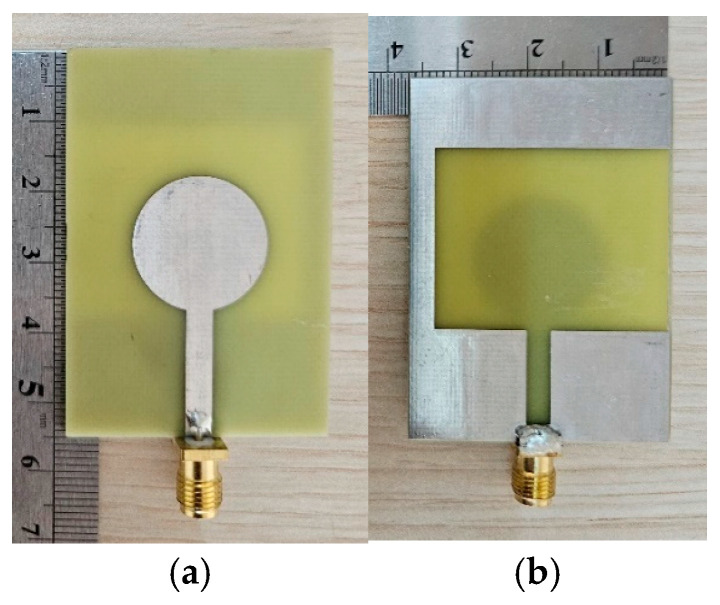
Fabricated antenna prototype: (**a**) top view showing the circular patch; (**b**) bottom view showing the slot-loaded ground plane.

To validate the simulation results, a prototype of the proposed antenna was fabricated using standard PCB techniques on an FR4 substrate with ε_r_ = 4.3 and a thickness of 1.6 mm. Both the circular patch on the top layer and the slot-loaded ground plane on the bottom layer are copper with 0.035 thickness. The fabricated antenna is shown in Figure 19.

**Figure 20:**
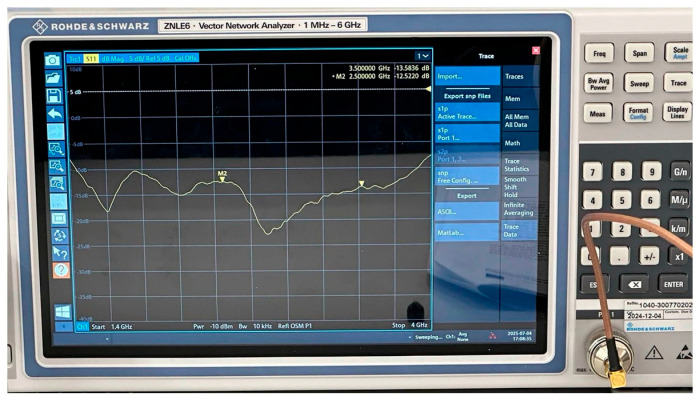
S_11_ measurement of the proposed antenna.

After fabrication of the antenna, the reflection coefficient (S_11_) was measured using a ZNLE6 vector network analyzer (Rohde & Schwarz, Munich, Germany) with a frequency range of 1.4–4 GHz, as seen in Figure 20.

**Figure 21:**
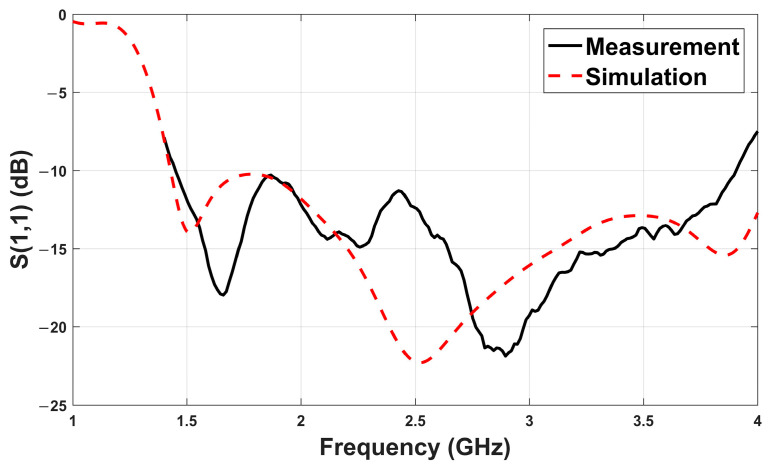
Comparison of simulated and measured S_11_ performance of the antenna.

Figure 21 compares the simulated and measured S_11_ responses. A slight frequency shift is observed in the measured results, which may be attributed to fabrication tolerances, soldering inconsistencies, or substrate losses. Nevertheless, both curves confirm that the antenna maintains a wide operating bandwidth, with S_11_ below –10 dB from approximately 1.43 to 4.0 GHz.

**Figure 22:**
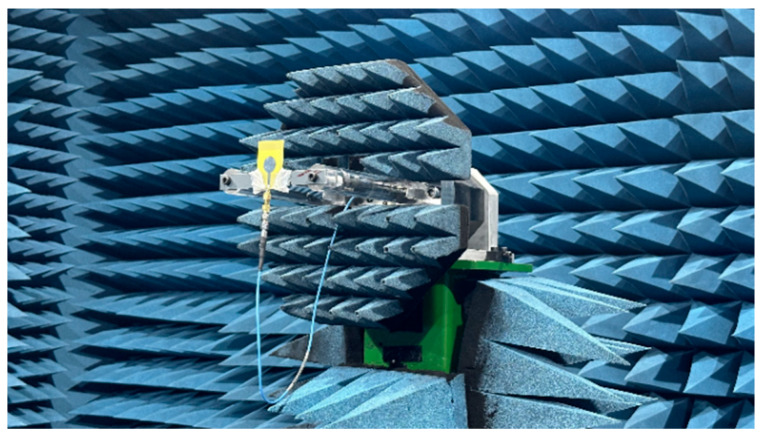
Radiation pattern measurement setup in the anechoic chamber.

To evaluate the radiation characteristics of the designed antenna, radiation pattern measurements were conducted in an anechoic chamber. The measurement setup of the radiation pattern of the proposed antenna is shown in Figure 22.

**Figure 23:**
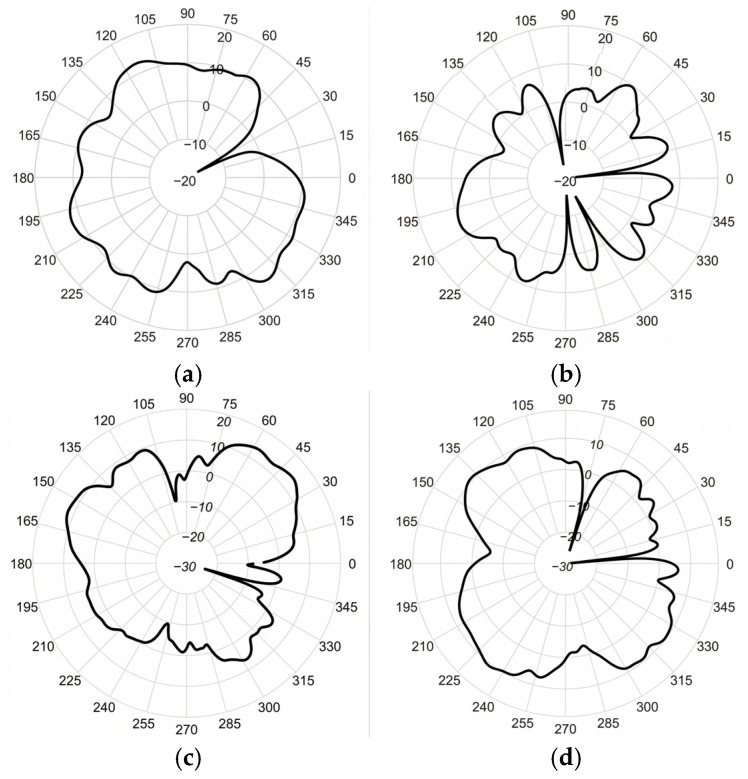
Measured radiation patterns of the proposed antenna on the E-plane at (**a**) 2.1 GHz; (**b**) 2.5 GHz; (**c**) 3.3 GHz; (**d**) 3.9 GHz.

Following the measurements, the radiation pattern of the proposed antenna on the E-plane was obtained, as presented in Figure 23.

The discrepancies between the measured and simulated radiation patterns in Figure 5 are primarily attributed to fabrication tolerances and measurement setup misalignments. Although minor distortions occur due to these practical constraints, the overall radiation characteristics remain generally consistent with the numerical results.

## 4. Discussion

As can be seen in Table 3, the proposed antenna design demonstrates a favorable balance between compact size, wideband performance, gain, fractional bandwidth, and fidelity factor, making it highly suitable for MWI applications, particularly in the detection and monitoring of brain tumors. Compared to existing works, it achieves a broad operational bandwidth of 1.43–4 GHz, which is the widest among all referenced designs, corresponding to a fractional bandwidth of 94.7%. This value is comparable to or exceeds that of many previously reported antennas, including larger designs operating on similar substrates. This confirms the strong wideband capability of the proposed structure. A high fractional bandwidth is particularly important in MWI systems, as it enables multi-frequency operation, improved penetration–resolution trade-offs, and enhanced contrast between healthy and malignant tissues. In addition, the proposed antenna exhibits a high fidelity factor of 95%, which is superior to or competitive with most existing designs reported in the literature. A high fidelity factor indicates minimal signal distortion during transmission and reception, ensuring that the radiated and received waveforms closely resemble the input signal. Despite its compact dimensions (37 × 54.5 × 1.6 mm^3^), the antenna maintains a realized gain of 3.7 dB, outperforming or closely matching several larger antennas listed in Table 3. Moreover, the use of a low-cost FR-4 substrate enhances its practicality for scalable and cost-effective medical applications, without significantly compromising performance.

It should be emphasized that the tumor localization results presented in this study are derived from simulation-based imaging scenarios, whereas the experimental validation is limited to electromagnetic characterization of the fabricated antenna. Consequently, measurement-based imaging experiments using realistic head phantoms will be an essential step for further validating the proposed system. In the current simulations, tumor responses are calibrated using a subtraction-based approach between healthy and tumor-bearing head models with identical structural dimensions. While this approach is feasible in a controlled simulation environment, it is not directly applicable to practical measurements. For realistic experimental and clinical implementations, more advanced signal-processing strategies will be necessary. In particular, adaptive filtering techniques—such as Wiener filtering [24] and the Root Least Squares Filter [25]—may be employed to isolate tumor responses without requiring an exact healthy reference model.

Collectively, the combination of wide fractional bandwidth, high fidelity factor, adequate gain, and compact size underscores the effectiveness of the proposed antenna as a robust and efficient candidate for non-invasive microwave brain tumor imaging systems.

## 5. Conclusions

This paper presents the design and simulation of a wideband circular microstrip patch antenna for radar-based MWI aimed at brain tumor detection. By introducing ground-plane slot modifications and performing structural optimization, the antenna achieves reliable impedance matching and directional radiation over the 1.43–4 GHz frequency band.

A voxel-based human head model is employed to simulate both tumor-present and tumor-absent scenarios. Background subtraction is used to isolate tumor-induced scattering, and the DAS beamforming algorithm is applied to reconstruct the dielectric distribution.

It is important to note that the tumor localization results presented in this work are based on numerical simulations, while experimental validation is limited to measurement and characterization of the fabricated antenna. Although experimental tumor localization using realistic head phantoms and tissue-mimicking materials is planned as future work, the simulation results demonstrate that the proposed system is capable of localizing high-permittivity inclusions with promising spatial resolution. Taken together, these findings highlight the strong potential of the proposed antenna and imaging approach for accurate, noninvasive brain tumor localization, and support the feasibility of this compact, low-cost system as a candidate for future clinical deployment, subject to further experimental validation.

## Figures and Tables

**Table 1:** Geometric parameters of the proposed antenna.

Parameters	W	L	Ls	Wf	r	Wsl	Lsl	Lfs	Wfs
Values (mm)	37	54.5	18.034	3.7	9.4	33.1	27	14	3.4

**Table 2:** Maximum SAR levels at the four different antenna positions and frequencies.

		Max SAR Level per 10 g w/kg			
Position	1.5 GHz	2.1 GHz	2.5 GHz	3.3 GHz	3.9 GHz
1	0.196	0.435	0.453	0.470	0.477
2	1.256	1.560	1.375	0.933	0.577
3	0.109	0.257	0.308	0.552	0.329
4	0.608	0.670	0.562	0.552	0.450

**Table 3:** Comparison of the proposed antenna to related works.

Reference	Size (mm^3^)	Substrate	Frequency (GHz)	Fractional Bandwidth (%)	Realized Gain (dB)	Fidelity Factor (%)
[6]	70 × 50 × 1.55	FR-4	0.80–1.20	40	Not Reported	Not Reported
[7]	37 × 56 × 1.6	FR-4	1.45–2.58	51.2	3.5	>80
[18]	70 × 60 × 1.5	FR-4	1.3–3.7	94.34	6.15	Not Reported
[19]	68.28 × 79 × 1.5	FR-4	1–2	66.67	Not Reported	Not Reported
[20]	60 × 70 × 1.6	FR-4	1.22–3.45	95.50	~6	92
[21]	38 × 30 × 1.6	FR-4	1–1.75	54.54	Not Reported	Not Reported
[22]	50 × 44 × 1.524	Rogers RO4350B (Rogers Corporation, Chandler, AZ, USA)	1.70–3.71	74.3	5.65	98
[23]	50 × 60 × 1.524	Rogers RO4350B	2.06–2.61	23.6	2.45	Not Reported
Proposed	37 × 54.5 × 1.6	FR-4	1.43–4	94.7	3.7	95

## Data Availability

Dataset available on request from the authors.

## Abbreviations

The following abbreviations are used in this manuscript:

CST	Computer Simulation Technology
CT	Computed Tomography
DAS	Delay and Sum
IEEE	The Institute of Electrical and Electronics Engineers
MRI	Magnetic Resonance Imaging
MWI	Microwave Imaging
SAR	Specific Absorption Rate
VNA	Vector Network Analyzer
